# Correlations of serum uric acid, fibrinogen and homocysteine levels with carotid atherosclerosis in hypertensive patients

**DOI:** 10.3389/fcvm.2025.1433107

**Published:** 2025-03-03

**Authors:** Liling Zhang, Shenlu Lu, Juanjuan Guo

**Affiliations:** Department of Geriatrics, Heping Hospital Affiliated to Changzhi Medical College, Changzhi, Shanxi, China

**Keywords:** serum uric acid, fibrinogen, homocysteine, hypertension, carotid atherosclerosis, carotid intima-media thickness, carotid atherosclerotic plaque area, receiver operating characteristic curve

## Abstract

**Objective:**

Uric acid (UA), fibrinogen (FIB), and homocysteine (Hcy) are the main contributors to cardiovascular and cerebrovascular diseases, and are related to hypertension. Hypertension plays a role in atherosclerosis (CAS). We hence explored the correlations of UA, FIB, and Hcy levels with CAS in hypertensive patients.

**Methods:**

Totally 170 hypertensive patients were retrospectively included and assigned into the Non-sclerosis, Thickened, and Plaque groups based on carotid intima-media thickness (cIMT), with serum UA, FIB, and Hcy compared. Correlations of UA, FIB, and Hcy with cIMT and carotid atherosclerotic plaque (CAP) were assessed using Spearman's correlation analysis. The risk factors of CAS were evaluated by logistic multivariate regression analysis. The predictive value of UA, FIB, and Hcy for CAS was estimated by the receiver operating characteristic (ROC) curve.

**Results:**

UA, FIB, and Hcy were up-regulated in the Plaque group vs. other two groups. Serum UA, FIB, and Hcy were positively linked to cIMT and CAP, and were independent risk factors for CAS. The area under ROC curve of UA, FIB, Hcy levels and their combination for predicting CAS were 0.889, 0.855, 0.902, and 0.958, respectively. Hypertensive patients with high levels of UA, FIB, or Hcy were more likely to develop CAS.

**Conclusion:**

Serum UA, FIB, and Hcy are positively correlated with cIMT and CAP, and are independent risk factors for CAS in hypertensive patients. High UA, FIB and Hcy expression could assist in predicting CAS in patients with hypertension, and the combination of the three was more valuable than all three alone.

## Introduction

Hypertension, which is defined as a persistent elevation in blood pressure levels equal to or exceeding 140/90 mmHg, is tied up with the development of cardiovascular disease and is widely prevalent in the general population (1). Hypertension is a condition that can be influenced by various factors, including lifestyle choices, dietary habits, and nutrient intake (2). Furthermore, hypertension is a predisposing factor for numerous cardiovascular diseases, thereby amplifying its importance as a risk factor (3, 4). Hypertension has now been acknowledged as the most significant risk factor for atherosclerosis. Carotid atherosclerosis (CAS) is a chronic, systemic condition and the primary cause of mortality in developed Western nations. With the development of China's economy and the alterations in lifestyle and dietary patterns, there has been a notable increase in the incidence of CAS, which has emerged as a critical contributor to mortality among middle-aged and elderly populations in China (4). CAS mainly refers to the gradual formation of carotid intima under the influence of various factors, and it initially presents as intima-media thickness, and gradually progresses to the formation of atherosclerotic plaques, which can rupture and become dislodged, leading to thrombosis and subsequent stenosis of the blood vessels, thereby triggering abnormal carotid artery blood supply (5).

Uric Acid (UA), fibrinogen (FIB) and homocysteine (Hcy) have been identified as significant contributors to the onset of cardiovascular diseases and may be linked with metabolic-related, such as hypertension (6–9). Serum UA has been extensively studied as a potential marker of inflammation and a risk factor for atherosclerosis, and UA metabolism plays a significant part in carotid plaque biology (10). UA has a direct impact on vascular function. The vascular endothelial cells express various UA transporters and incorporated UA impairs the production of nitric oxide, resulting in endothelial dysfunction (11). FIB is an essential coagulation factor that exerts a substantial influence on the progression of atherosclerosis via its ability to promote the proliferation of smooth muscle cells (12). FIB has been positively related with the prevalence and incidence of cardiovascular disease, primarily through mechanisms involving hyperviscosity and thrombosis in response to inflammatory processes. FIB has been reported as an independent predictor for early atherosclerotic disease, such as increased carotid intima–media wall thickness (cIMT) (13). Hcy, a byproduct of methionine methylation, possesses the capacity to disturb the regular functioning of vascular endothelial cells when there is an imbalance in its secretion, and this disruption can result in inflammatory reactions at the site of vascular injury, promoting the proliferation of smooth muscle cells, and ultimately contributing to the development of atherosclerosis (14). Furthermore, the serum UA level has been suggested to correlate with FIB and Hcy levels (15). It has been documented that the occurrence of CAS in patients with epilepsy has something to do with UA, FIB and Hcy (16). Nevertheless, the correlations between serum UA, FIB and Hcy levels with CAS in hypertensive patients has been scarcely investigated and require further investigation. Hence, the present study aimed to examine the correlations between serum UA, FIB and Hcy levels with CAS in individuals with hypertension to establish a reference range that could be utilized for predicting the severity of CAS in hypertensive patients. The primary finding of our study is that high expression of UA, FIB, and Hcy could assist in predicting CAS in hypertensive patients, with the combination of the three more valuable than all three alone. Our secondary findings indicated that serum UA, FIB, and Hcy were positively correlated with cIMT and CAP, and were independent risk factors for CAS. We propose that these findings may offer novel insights for the clinical diagnosis of CAS in hypertensive patients.

## Subjects and methods

### Ethics statements

The study was conducted in compliance with the recommendations set forth by the Academic Ethics Committee of Heping hospital affiliated to Changzhi Medical College [No. (2024)011]. All procedures were performed abided by the Declaration of Helsinki strictly.

### Study subjects

A minimum of 159 samples were required for this study, as calculated using G*Power 3.1.9.7 software (Supplementary Figure S1). A total of 170 hypertensive patients visited the geriatric outpatient clinic of Heping hospital affiliated to Changzhi Medical College from January 2020 to December 2022 were retrospectively included in this study based on the inclusion and exclusion criteria (Supplementary Figure S2). The study participants were then assigned into the Non-sclerosis group (*n* = 53) (patients whose carotid arteries were not yet sclerosed), the Thickened group (*n* = 57) (patients whose carotid arteries were thickened but no carotid atherosclerotic plaques were produced) and the Plaque group (*n* = 60) (patients who developed carotid atherosclerotic plaques) according to cIMT. The diagnosis and grouping of hypertension were conducted in accordance with the 2018 Chinese Guidelines for the Prevention and Treatment of Hypertension.

### Inclusion and exclusion criteria

The inclusion criteria encompassed the hypertension patients who were with systolic blood pressure (SBP) ≥ 140 mmHg or diastolic blood pressure (DBP) ≥ 90 mmHg, underwent carotid artery ultrasonography and biochemical examination, and with complete clinical information.

The exclusion criteria included patients with pulmonary hypertension or autoimmune disease or complicated with other carotid artery diseases, vegetarians, patients who had recently consumed medications or foods that affected homocysteine level, such as folic acid, vitamins, and betaine, or patients who received proton pump inhibitor treatment in the past six months.

Hypertensive patients were grouped as per the following criteria: the Non-sclerosis group (cIMT < 1.0 mm); the Thickened group (1.0 mm ≤ cIMT ≤ 1.50 mm); the Plaque group (cIMT > 1.50 mm).

In the light of the Echocardiography Guidelines and Standards of the American Society (17), CAS was diagnosed as that carotid arteries exhibited limited or diffuse intima-media thickening, generally greater than 1.5 mm, along with additional atherosclerotic plaques (18–20).

### Data collection

Clinical data such as age, sex, body mass index (BMI), disease course, smoking history, drinking history, diabetes history, SBP, DBP, carotid atherosclerotic plaque (CAP) and cIMT, as well as biochemical data such as triacylglycerols (TG), total cholesterol (TC), UA, FIB and Hcy were acquired via the integrated hospital information system such as the outpatient medical record system and inspection system. All patients were instructed to fast for at least 12 h before venous blood collection. Elbow vein blood (9 mL) was collected in EDTA-K2 anticoagulated vacuum tubes and placed for 30 min. After centrifugation, the samples were preserved at −80°C for measurement. Serum levels of TG, TC, UA, FIB and Hcy were measured using a fully automatic biochemical analyzer (PUZS-300, TIPS, Shanghai, China).

### Imaging examination

cIMT was determined by Doppler color ultrasound (Vivid E95, GE, Boston, MA, USA) using a b-mode 12 MHz linear probe. All subjects were examined by a dedicated full-time ultrasonographer. The patients were positioned supine, with the neck fully extended by elevating the shoulders and slightly tilting the head backward. For the examination of one side, the head was tilted to the contralateral side. The ultrasound probe was positioned at the sternoclavicular joint, medial to the sternocleidomastoid muscle, with the sound beam oriented posteriorly. Both the long axis and short axis of the carotid artery were scanned. The detection range extended 1 cm above the initial site of the internal carotid artery. The thickness of the arterial wall, the presence or absence of plaque formation, the morphology and dimensions of the plaque, as well as the presence or absence of stenosis were observed. cIMT was defined as the distance between the intima-lumen interface and the medial-adventitia interface. The test was performed at the thickest point 1 cm away from the proximal segment of the bifurcation of the common carotid artery. Measurements were taken three times for both the left and right sides, with the left and right cIMT values calculated as the average of these three measurements. The final cIMT value was subsequently determined based on the average of the left and right cIMT values.

### Establishment of logistic regression model

Logistic multivariate regression was utilized to analyze the independent risk factors for CAS. BMI, age, sex, disease course, drinking history, smoking history, diabetes history, SBP, DBP, and TG, TC, UA, FIB, and Hcy levels were included as covariates, and the development of CAS as the dependent variable. The values of “Plaque group” = 1, “Non-sclerosis group” and “Thickened group” = 0 were assigned. Based on the results of factor analysis, the regression outcomes were further examined using stepwise regression analysis, which controlled for the range of independent variables, to ensure the robustness of the regression findings.

### Data analysis

SPSS 25.0 (IBM, Armonk, New York, USA) and GraphPad Prism 6.0 (GraphPad, San Diego, CA, USA) were applied for data analysis. The normality of measurement data was evaluated by Shapiro–Wilk test. Continuous variables that conformed to normal distribution were presented as mean ± standard deviation, with one-way analysis of variance (ANOVA) for comparisons among groups and Tukey's multiple comparison test for *post hoc* analysis. For data that did not conform to a normal distribution, results were expressed as median (minimum, maximum), and comparisons among groups were conducted using the Kruskal–Wallis test. Categorical variables were reported as number of cases and percentage (%), with the *χ*^2^ test for inter-group comparisons. Spearman correlation analysis was implemented to assess the correlations of UA, FIB and Hcy with cIMT and plaque score. The predictive value of UA, FIB, and Hcy for CAS was assessed using receiver operating characteristic (ROC) curve. *P* value was calculated using a two-sided test, and differences were considered statistically significant at *P* < 0.05.

## Results

### Clinical baseline characteristics of study subjects

No prominent differences in age, sex, BMI, and drinking history were observed among the Non-sclerosis, Thickened, and Plaque groups (all *P* > 0.05), while there were significant differences in smoking, SBP, DBP, TG, cIMT, and CAP in the three groups (Non-sclerosis group < Thickened group < Plaque group) (all *P* < 0.05). Disease course and TC did not exhibit significant differences between the Non-sclerosis group and the Thickened group (all *P* > 0.05), whereas significant differences in disease course and TC were observed in the Plaque group when compared to both the Non-sclerosis and Thickened groups (all *P* < 0.05, Table 1).

**Table 1:** Comparisons of clinical baseline characteristics.

Baseline characteristics		Non-sclerosis group (*n* = 53)	Thickened group (*n* = 57)	Plaque group (*n* = 60)	*P*_a_	*P*_b_	*P*_c_	*P*_d_
Age (year)		47 (41–56)	47 (42–57)	48 (43–55)	>0.99	0.12	0.18	0.07
Sex	Male	27	29	30	>0.99	>0.99	>0.99	0.99
	Female	26	28	30				
BMI (kg/m^2^)		23.07 (20.04–25.98)	23.03 (20.11–26.00)	23.21 (18.52–25.94)	>0.99	0.85	0.74	0.43
Disease course (years)		4 (0.18–12.26)	4.27 (0.94–8.05)	6.32 (1.91–12.26)	>0.99	<0.001	<0.001	<0.05
Drinking history (cases)		17	19	21	>0.99	>0.99	>0.99	0.95
Smoking history (cases)		10	30	46	<0.001	<0.001	<0.001	<0.05
Diabetes history (cases)		8	18	33	<0.05	<0.05	<0.05	<0.05
SBP (mmHg)		154.6 ± 6.396	162.6 ± 7.843	175.1 ± 10.43	<0.001	<0.001	<0.001	<0.001
DBP (mmHg)		98.72 (90.36–108.90)	101.40 (91.43–128.2)	103.7 (95.12–124.4)	<0.01	<0.05	<0.001	<0.001
TG (mmol/L)		1.17 (0.09–5.96)	1.47 (0.68–6.84)	2.13 (0.04–6.33)	<0.05	<0.05	<0.01	0.04
TC (mmol/L)		3.98 (0.56–10.79)	4.85 (1.41–8.74)	6.96 (5.44–8.86)	0.15	<0.001	<0.001	<0.01
cIMT (mm)		0.81 (0.51–0.99)	1.23 (1.03–1.49)	1.68 (1.50–2.16)	<0.001	<0.001	<0.001	<0.05
CAP (mm^2^)		4.62 ± 1.87	7.86 ± 1.38	12.09 ± 1.08	<0.001	<0.001	<0.001	<0.001
Antihypertensive drugs (cases)								
Diuretics		4	2	3	0.533	0.639	0.294	0.639
Calcium channel blockers		32	34	38				
Beta-blockers		6	8	4				
Angiotensin II receptor blockers		11	15	18				
Angiotensin converting enzyme inhibitors		5	5	9				

Note: *P*_a_: Non-sclerosis group vs Thickened group; *P*_b_: Thickened group vs Plaque group; *P*_c_: Non-sclerosis group vs Plaque group; *P*__d__: Comparisons among the Non-sclerosis, Thickened and Plaque groups. Counting data were expressed as number of cases and percentage, and comparisons between groups were made using Chi-square test. Normally distributed measures were expressed as mean ± standard deviation and tested by one one-way ANOVA. Non-normally distributed measures were expressed as median (minimum value, maximum value), and comparisons between groups were made utilizing the Kruskal–Wallis test. BMI, body mass index; SBP, systolic blood pressure; DBP, diastolic blood pressure; TG, triacylglycerols; TC, total cholesterol; cIMT, carotid intima-media thickness; CAP, carotid atherosclerotic plaque.

### Comparisons of serum UA, FIB and Hcy levels in the three groups of hypertensive patients

**Figure 1:**
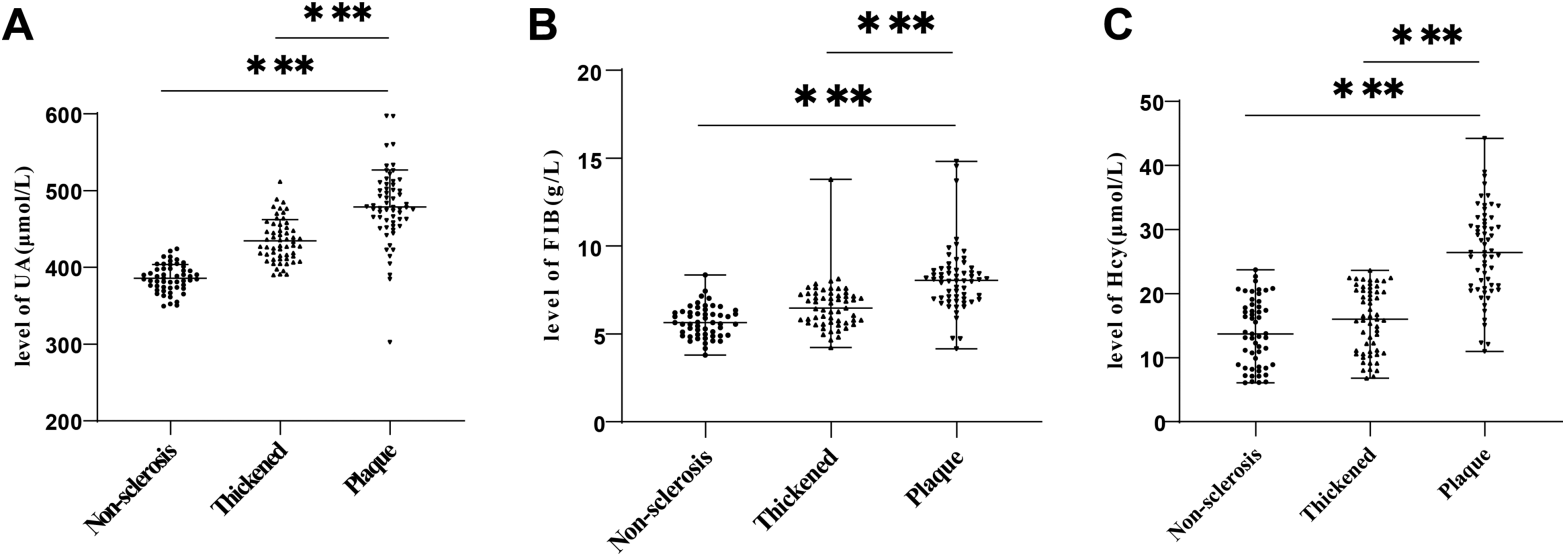
Comparisons of UA, FIB and Hcy levels in the three groups of hypertensive patients. **(A)** Comparison of UA levels; **(B)** Comparison of FIB levels; **(C)** Comparison of Hcy levels. Inter-group comparisons of normally distributed measurement data were conducted using one-way ANOVA. Inter-group comparisons of measurement data with non-normal distribution were performed using Kruskal–Wallis test. *** *P* < 0.001. UA, Uric acid; FIB, fibrinogen; Hcy, homocysteine.

We compared serum UA, FIB and Hcy levels in three groups, with the results showing that serum UA, FIB and Hcy levels in the Non-sclerosis group were (385.74 ± 17.10) *μ*mol/L, [5.64 (3.80–8.36)]g/L, [13.72 (6.06–23.70)] *μ*mol/L, respectively, those in the Thickened group were (434.62 ± 27.56) *μ*mol/L, [6.48 (4.23–13.80)] g/L, and [16.00 (6.79–23.64)] *μ*mol/L, and those in the Plaque group were (478.78 ± 48.23) *μ*mol/L, [8.05 (4.16–14.82)] g/L, and [26.43 (10.96–44.24)] *μ*mol/L, respectively. These results demonstrated that UA, FIB and Hcy levels were distinctly elevated in the Plaque group relative to the Non-sclerosis and Thickened groups (Figures 1A–C, all *P* < 0.001).

### Correlations of serum UA, FIB and Hcy levels with cIMT and CAP in hypertensive patients

**Figure 2:**
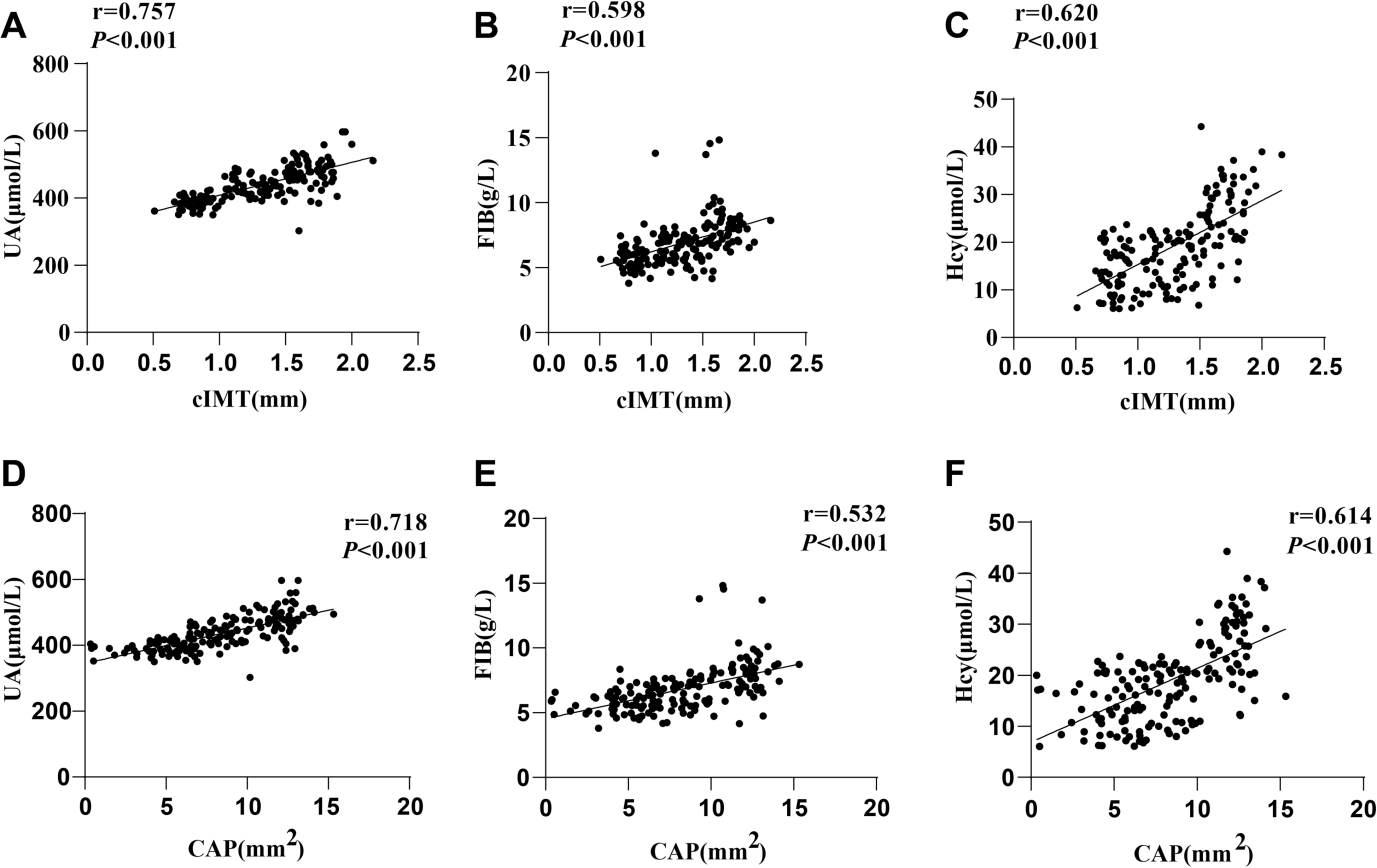
Correlation analyses. Spearman's correlation coefficient was used to analyze the correlations of cIMT with **(A)** UA, **(B)** FIB, and **(C)** Hcy, as well as the correlations of CAP with **(D)** UA, **(E)** FIB, and **(F)** Hcy. UA, Uric acid; FIB, fibrinogen; Hcy, homocysteine.

There were significant differences in serum UA, FIB and Hcy levels among the three groups of hypertensive patients. Spearman's correlation coefficient was conducted to assess the correlations of UA, FIB and Hcy with CAS markers (cIMT and CAP), with the results showing that cIMT was favorably lined with UA (*r* = 0.757, *P* < 0.001), FIB (*r* = 0.598, *P* < 0.001) and Hcy (*r* = 0.620, *P* < 0.001) levels (Figures 2A–C), and CAP was positively interrelated with UA (*r* = 0.718, *P* < 0.001), FIB (*r* = 0.532, *P* < 0.001) and Hcy (*r* = 0.614, *P* < 0.001) levels (Figures 2D–F).

### Serum UA, FIB, and Hcy levels were independent risk factors for CAS in hypertensive patients

To probe whether UA, FIB and Hcy were risk factors for CAS, CAS was included as the dependent variable, and the indicators with *P* < 0.05 in Table 1 and Figure 1 were included as independent variables in the logistic univariate regression analysis model one by one. The results unveiled that disease course, smoking, diabetes, SBP, DBP, TG, TC, UA, FIB and Hcy levels were independent risk factors for CAS (all *P* < 0.05, Table 2). After adjusting for confounding variables and performing multicollinearity analysis (Table 3), variables with *P* < 0.05 and variance inflation factor (VIF) values <10 were incorporated in the logistic multivariate regression analysis model. As reflected by the results, SBP, DBP, TG, TC, UA, FIB, and Hcy levels were independent risk factors for CAS (all *P* < 0.05, Table 2).

**Table 2:** CAS risk factor analyses.

Factors	Logistic multivariate analysis			Logistic univariate analysis		
	*P* value	*OR* value	95% CI	*P* value	*OR* value	95% CI
Age	0.128	1.068	0.981, 1.163	–	–	–
Sex	0.91	0.964	0.514, 1.809	–	–	–
BMI	0.501	1.115	0.812, 1.531	–	–	–
Disease course	<0.001	1.975	1.603, 2.434	0.253	1.437	0.772, 2.676
Smoking	<0.001	4.183	2.037, 8.588	0.286	0.299	0.033, 2.749
Drinking history	0.764	1.107	0.570, 2.149	–	–	–
Diabetes	<0.001	3.949	2.016, 7.736	0.057	5.006	0.953, 26.282
SBP	<0.001	1.211	1.143, 1.282	<0.001	1.293	1.165, 1.435
DBP	<0.001	1.158	1.085, 1.236	0.001	1.189	1.071, 1.320
TG	0.001	1.519	1.174, 1.965	<0.001	2.875	1.640, 5,040
TC	<0.001	2.74	1.952, 3.845	<0.001	3.298	1.818, 5.982
cIMT	0.928	1.235	0.013, 115.699	–	–	–
UA	<0.001	1.042	1.029, 1.055	0.012	1.022	1.005, 1.040
FIB	<0.001	2.972	2.068, 4.273	<0.001	2.295	1.523, 3.458
Hcy	<0.001	1.399	1.256, 1.559	<0.001	1.353	1.173, 1.562

Note: BMI, body mass index; SBP, systolic blood pressure; DBP, diastolic blood pressure; TG, triacylglycerols; TC, total cholesterol; UA, Uric acid; FIB, fibrinogen; Hcy, homocysteine.

**Table 3:** Multicollinearity analysis.

Dependent variable VIF Variable	SBP	DBP	TG	TC	UA	FIB	Hcy
SBP	–	2.863	2.693	2.805	2.457	2.831	2.301
DBP	1.147	–	1.131	1.134	1.125	1.124	1.144
TG	1.133	1.187	–	1.201	1.201	1.197	1.057
TC	1.274	1.286	1.297	–	1.297	1.274	1.296
UA	2.308	2.637	2.682	2.682	–	2.63	2.346
FIB	1.342	1.33	1.349	1.329	1.327	–	1.356
Hcy	2.291	2.842	2.501	2.839	2.486	2.849	–

Note: VIF, Variance Inflation Factor; SBP, systolic blood pressure; DBP, diastolic blood pressure; TG, triacylglycerols; TC, total cholesterol; UA, Uric acid; FIB, fibrinogen; Hcy, homocysteine. acid; FIB, fibrinogen; Hcy, homocysteine.

### Serum UA, FIB and Hcy levels helped predict CAS in hypertensive patients

**Figure 3:**
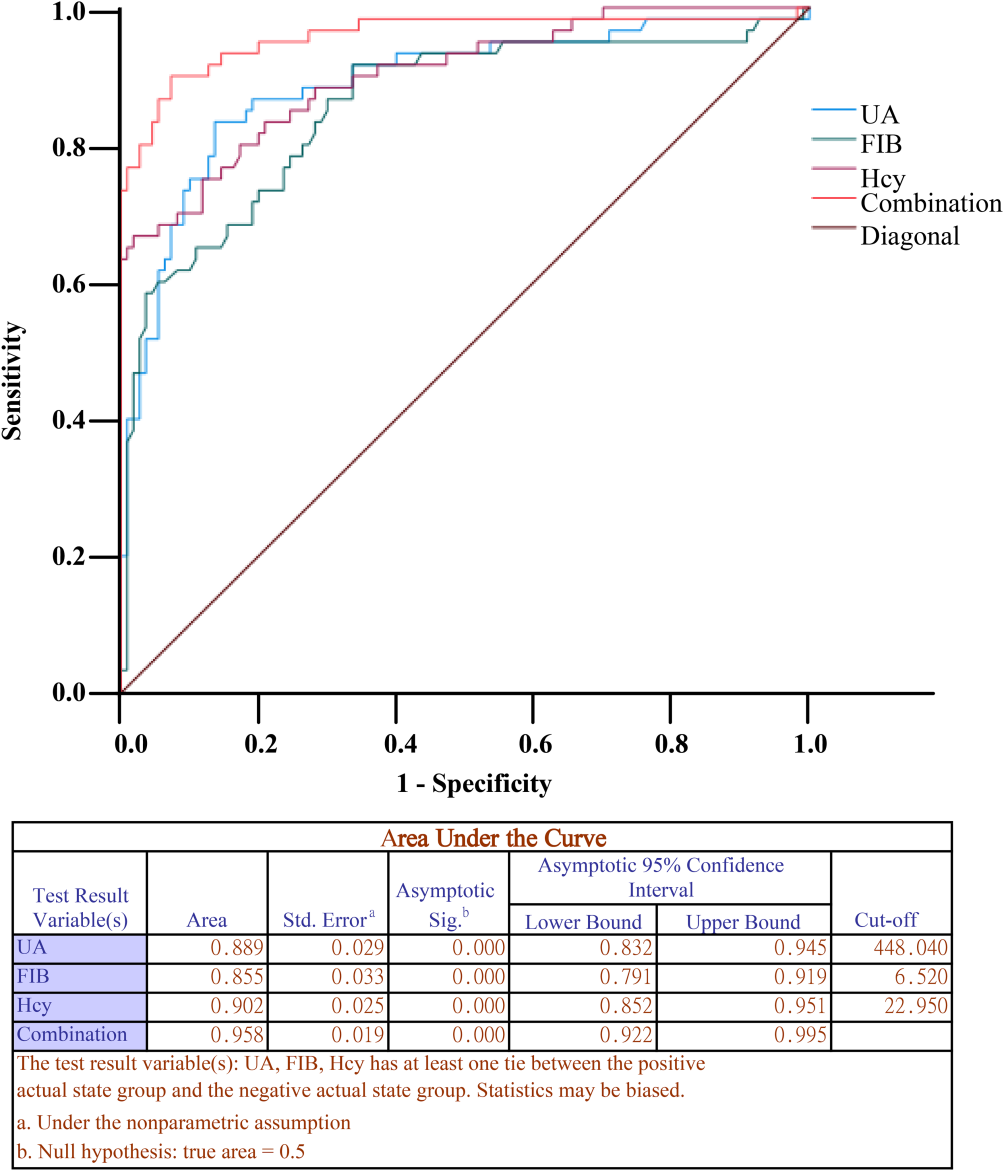
ROC curve. ROC curves were plotted to assess the predictive value of UA, FIB, and Hcy levels for CAS. UA, Uric acid; FIB, fibrinogen; Hcy, homocysteine.

ROC curves were plotted to assess the predictive value of UA, FIB and Hcy levels for CAS. As listed in Table 4 and Figure 3, area under the curve (AUC) for the UA level to predict CAS was 0.889 (sensitivity of 83.33%, specificity 86.36%, and cut-off value of 448.04 *μ*mol/L), that for the FIB level was 0.855 (sensitivity 91.67%, specificity 66.36%, cut-off value 6.52 g/L), and that for the Hcy level was 0.902, with the sensitivity 66.67%, specificity 98.18%, and cut-off value 22.95 *μ*mol/L. Subsequently, ROC curves were plotted to analyze the predictive value of combination of serum UA, FIB, and Hcy levels in hypertensive patients (a diagnostic panel model for UA, FIB, and Hcy was established, referred to as Combination) for CAS. It was found that the combination of UA, FIB and Hcy levels had an AUC of 0.958 (optimal sensitivity 90.00%, specificity 92.73%), indicating that the predictive value of the combination of the three factors was higher than that of each factor alone (all *P* < 0.01). Additionally, the patients were arranged into the low/high expression groups based on the cutoff values of UA, FIB and Hcy levels to compare the incidence of CAS. As shown in Table 5, highly-expressed UA, FIB or Hcy was more likely to lead to CAS.

**Table 4:** Parameters of the ROC curve.

	AUC	95% CI	Sensitivity	Specificity	*P*_a_	*P*_b_	*P*_c_	*P*_d_
UA	0.889	0.832–0.945	83.33%	86.36%	<0.001	0.003	<0.001	0.004
FIB	0.855	0.792–0.919	91.67%	66.36%	<0.001			
Hcy	0.902	0.852–0.951	66.67%	98.18%	<0.001			
Combination	0.958	0.922–0.995	90.00%	92.73%	<0.001			

Note: *P*_a_: ROC significance level P; *P*_b_: UA vs Combination; *P*_c_: FIB vs Combination; *P*_d_: Hcy vs Combination. The analysis was performed by ROC analysis, and comparisons among the multiple AUCs were conducted using the Delong test in MEDCALC software. UA, Uric acid; FIB, fibrinogen; Hcy, homocysteine.

**Table 5:** CAS in hypertensive patients with different levels of UA, FIB and Hcy.

	Patients without CAS	Patients with CAS	*P* value
UA Low expression	95	9	*P* < 0.001
UA high expression	15	51	
FIB low expression	73	4	*P* < 0.001
FIB high expression	37	56	
Hcy low expression	108	20	*P* < 0.001
Hcy high expression	2	40	

Note: The count data were expressed as the number of cases, and the chi-square test was conducted for comparison between groups. UA, Uric acid; FIB, fibrinogen; Hcy, homocysteine.

## Discussion

Hypertension is not solely a risk factor for cerebral, heart, and kidney diseases, but also serves as an autonomous risk factor for atherosclerosis (21, 22). Hypertension and atherosclerosis are widely recognized as the leading causes of mortality in both rich and poor nations (23). CAS is a significant manifestation of atherosclerosis, which is primarily bound up with the onset of stroke, and there are no specific preventive measures for CAS, apart from the implementation of established cardiovascular risk reduction strategies (24). Therefore, it is imperative to identify and implement efficacious strategies for CAS. Our findings highlighted that elevated levels of UA, FIB and Hcy might serve as predictive markers for CAS in hypertensive patients. Furthermore, the combined assessment of all these three biomarkers demonstrated greater predicative value compared to their individual evaluation.

Serum UA has been found to be correlated with various cardiovascular risk factors, including subclinical atherosclerosis and metabolic syndrome (25–27). Elevated level of UA have been recognized as a significant risk factor for conditions such as hypertension, and it has been observed that fluctuating UA level can not only trigger inflammation in the coronary arteries, but also plays a role in the progression of atherosclerosis (28). UA level exhibits positive correlations with BMI, TC, TG, SBP, DBP, and is independent risk factors for CAS in patients diagnosed with T2DM (29). FIB, as an inflammatory marker, plays a direct part in the development of atherosclerosis (30). It has been discovered that interaction between FIB and P-selectin pitches in the progression of atherosclerosis, and enhanced FIB level is a potential risk factor for atherosclerotic biomarkers (31). Another key focus of our study, Hcy is also recognized as an autonomous risk factor for atherosclerosis, a condition featured by the buildup of lipids within atherosclerotic plaques (32). It has been documented to expedite the progression of atherosclerosis and elicit vascular inflammation through the upregulation of inflammatory genes (33). A recent study has also unraveled that elevated serum Hcy level is tightly tied up with the severity of CAS (34). In this study, comparisons were made in terms of the levels of serum UA, FIB and Hcy among the three distinct groups of hypertensive patients, with the results showing that the levels of UA, FIB, and Hcy were markedly elevated in the Plaque group when compared to the Non-sclerosis and Thickened groups. Moreover, the multivariate regression model was used to identify independent risk factors for CAS. After adjusting for possible confounders such as age, sex, obesity, and diabetes history (35–37) and eliminating the effects of multicollinearity, logistic multivariate regression analysis results revealed that SBP, DBP, TG, TC, UA, FIB and Hcy levels were independent risk factors for CAS.

Ultrasound examination of the carotid arteries to determine cIMT and CAP predicts the risk of cardiovascular disease, and as a proxy for atherosclerotic disease, CAP contributes to approximately 20% of the risk associated with stroke and coronary artery diseases (38, 39). A recent study has demonstrated a pronounced correlation between serum UA level and cIMT (40). FIB level exhibits a favorable correlation with maximal cIMT, and there is an increased prevalence of carotid plaque in patients with raised FIB level (41). Hcy level is found to be significantly elevated in hypertensive patients with CAP compared to those without plaque, and elevated plasma Hcy level may play a role in the development of CAS (42). We conducted a Spearman correlation analysis to assess the relationships between UA, FIB and Hcy levels with the markers of CAS (cIMT and CAP). The findings indicated positive correlations between cIMT and the levels of UA, FIB and Hcy. Additionally, there were positive correlations between CAP and the levels of UA, FIB and Hcy. Elevated levels of UA, FIB, or Hcy were found to be associated with an increased risk of CAS. Subsequently, the predictive value of serum UA, FIB, or Hcy levels for CAS was assessed by plotting a ROC curve. The results revealed that the combined measurement of UA, FIB, and Hcy levels exhibited a greater predictive value for CAS compared to the individual measurements of these three biomarkers alone.

Although our findings indicated that UA, FIB, and Hcy levels in hypertensive patients were predictive of CAS, the dynamic changes in these biomarkers warranted further investigation. Many studies have explored the effect of antihypertensive medications on UA, FIB, and Hcy levels. For example, long-term use of diuretics has been linked to the development of hyperuricemia (43). Antihypertensive therapy utilizing calcium channel blockers has been associated with a reduction in the serum UA level (44). Angiotensin II receptor blockers have demonstrated efficacy in lowering the serum UA level in hypertensive patients (45). Diuretics, beta-blockers and alpha-1 blockers decrease glomerular filtration rate, while simultaneously elevating the serum UA level. In contrast, calcium channel blockers, angiotensin-converting enzyme inhibitors, and angiotensin receptor blockers, including losartan, do not appear to increase serum UA levels (46). However, it has been documented that among the five angiotensin II receptor blockers, only losartan significantly reduces serum UA levels within one year (47). Angiotensin II receptor blockers, including losartan and candesartan, have been shown to down-regulate FIB levels in hypertensive patients (48). A prior study has reported that the combination of angiotensin converting enzyme inhibitor delapril and the calcium channel blocker manidipine results in a reduction in the FIB level in hypertensive patients (49). Diuretics up-regulate the Hcy level, whereas beta-blockers and angiotensin converting enzyme inhibitors down-regulate Hcy in hypertensive patients (50). Additionally, long-term administration of calcium channel blockers has been found to remarkably diminish Hcy levels in hypertensive patients (51). The alterations in UA, FIB, and Hcy levels resulting from antihypertensive medications may introduce potential confounding effects on our findings.

Taken together, our study highlighted that elevated levels of UA, FIB and Hcy might serve as predictive markers for CAS in hypertensive patients. Furthermore, the combined assessment of all these three biomarkers demonstrated greater prognostic value compared to their individual evaluation. However, this article also had several important limitations to consider. First, the limited number of cases and events, as well as its single-center design, constrained the cross-sectional design. Both female sex and menopausal status have been shown to influence levels of UA, FIB and Hcy in hypertensive patients. Specifically, premenopausal estrogen level in women may enhance enhance renal clearance of urate, while serum UA concentrations are, on average, approximately 1 mg/dl higher in adult men compared to women (52). Additionally, FIB level is consistently elevated in women relative to men and tend to increase following menopause, which is associated with hormone deficiency in menopause elderly women (53, 54). Furthermore, plasma Hcy level is lower in women of childbearing age relative to their male counterparts and postmenopausal women, with the heightened risk of cardiovascular disease in postmenopausal women being linked with elevated Hcy levels (55). However, due to the limitations of single-centre and small sample size of this study, it was not possible to conduct separate analyses based on sex or menopausal status. Secondly, this study did not analyze the comorbidity of hyperhomocysteinemia in hypertensive patients different sexes nor did it explore the correlation between different Hcy subgroups and CAS, such as in patients with mild to moderate hyperhomocysteinemia. Thirdly, a related study examining patients with moderate or mild hyperhomocysteinemia found that the risk factor for CAS in patients with moderate hyperhomocysteinemia could be elevated to 1.4 (56), suggesting that varying degrees of hyperhomocysteinemia exert distinct influences on the onset and progression of CAS. Fourthly, our analysis on potential confounders, including the administration of antihypertensive medication, dietary habits, socioeconomic status, and genetic predisposition, was not comprehensive enough. As indicated in the previous studies, vegetarians display a lower level of serum high-density lipoprotein than individuals adhering to conventional diets. Moreover, vegetarians show an improved ability to combat atherosclerosis through the supplementation of vitamin B or folic acid (9, 57). Untreated hypertensive men residing in deprived neighborhoods are more likely to develop preclinical CAS compared to their counterparts living in affluent areas (58). Genetic predispositions also play a significant role in the incidence of CAS. For instance, The variant associated with coronary heart disease located at the 10q11.21 locus has been linked to cIMT and atherosclerosis (59). Consequently, it is imperative to extend the analysis of CAS occurrence among hypertensive patients in different Hcy subgroups to gain deeper insights into the clinical implications of Hcy level on CAS in hypertensive patients. In future research, we intend to expand the sample size and conduct a multicenter study to improve the understanding of the predictive capacity of UA, FIB and Hcy for hypertensive patients regarding CAS. Additional patient data will be collected and include incorporated into the analyses to minimize the influence of confounding factors that may impact the study's results. Additionally, we will further refine the clinical data through telephone follow-ups and analyze the effects of lipid-lowering therapy in subsequent studies.

## Data Availability

The data that support the findings of this study are available from the corresponding author upon reasonable request.

## Supplementary material

Supplementary Figure S1 The minimum sample size was calculated using G*Power software.

Supplementary Figure S2 Flowchart.
