# Supplementary figures and images for: Correlations of serum uric acid, fibrinogen and homocysteine levels with carotid atherosclerosis in hypertensive patients
Source: Front Cardiovasc Med. 2025 Mar 3;12:1433107. doi: 10.3389/fcvm.2025.1433107 (PMC11911491; doi:10.3389/fcvm.2025.1433107)

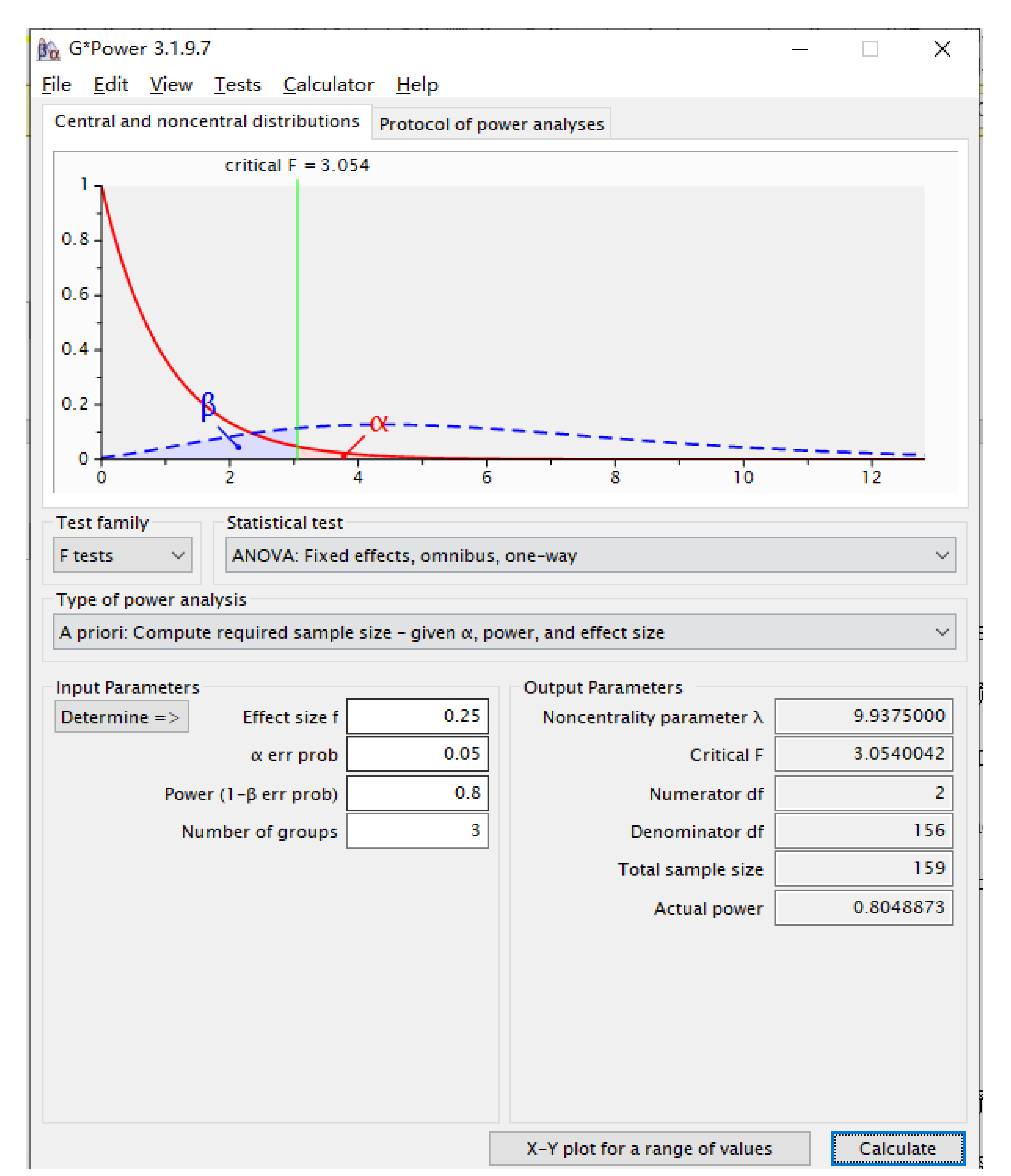

Supplement: Supplementary Figure S1 [file Image1.tiff]

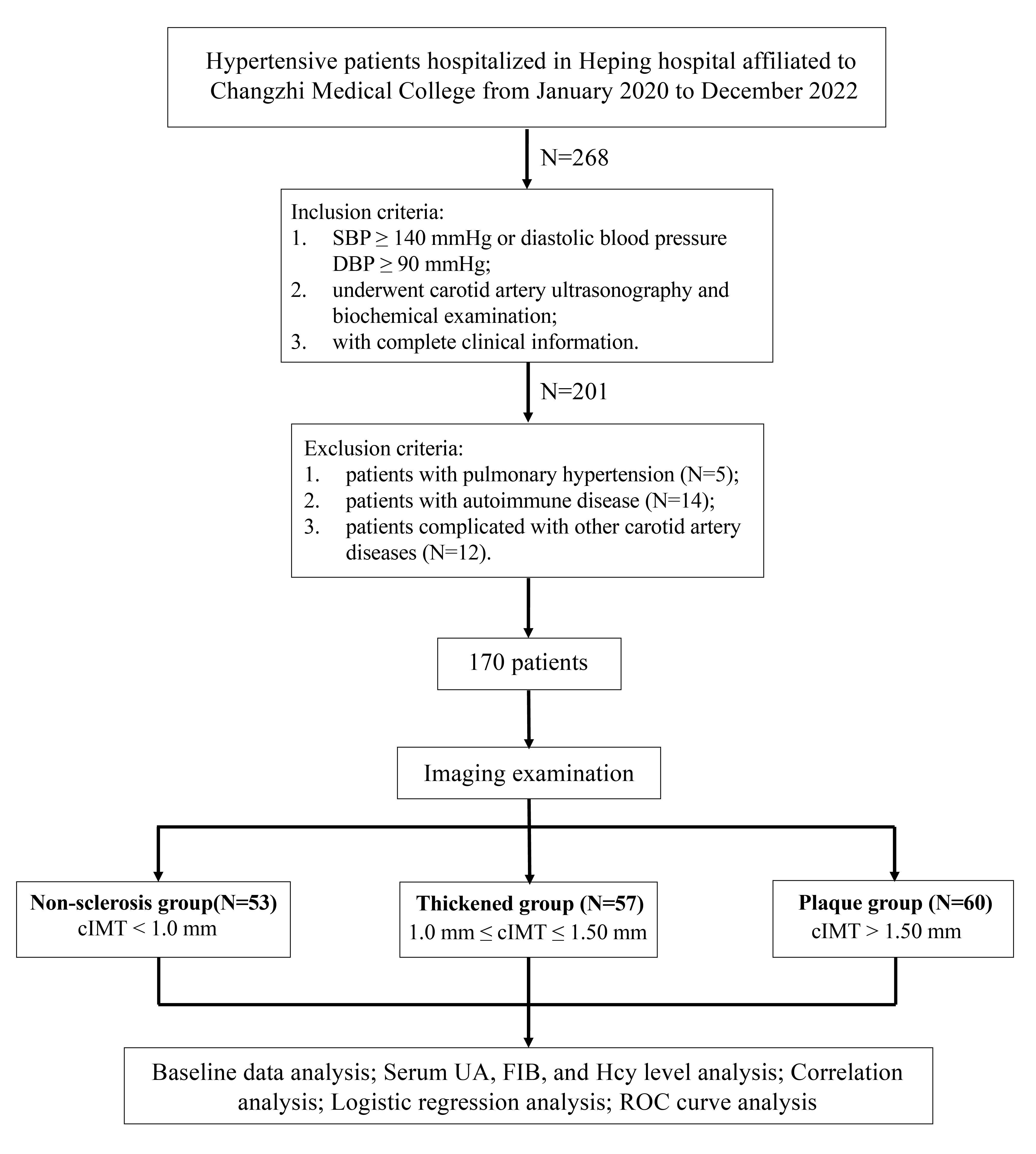

Supplement: Supplementary Figure S2 [file Image2.tiff]
